# Histologically Confirmed Recellularization is a Key Factor that Affects Meniscal Healing in Immature and Mature Meniscal Tears

**DOI:** 10.3389/fcell.2021.793820

**Published:** 2021-12-08

**Authors:** Wenqiang Yan, Wenli Dai, Jin Cheng, Yifei Fan, Fengyuan Zhao, Yuwan Li, Maihemuti Maimaitimin, Chenxi Cao, Zhenxing Shao, Qi Li, Zhenlong Liu, Xiaoqing Hu, Yingfang Ao

**Affiliations:** ^1^ Department of Sports Medicine, Peking University Third Hospital, Beijing, China; ^2^ Institute of Sports Medicine of Peking University, Beijing, China; ^3^ Beijing Key Laboratory of Sports Injuries, Beijing, China

**Keywords:** meniscal tear, meniscal repair, recellularization, immature menisci, mature menisci, extracellular matrix

## Abstract

Healing outcomes of meniscal repair are better in younger than in older. However, exact mechanisms underlying superior healing potential in younger remain unclear from a histological perspective. This study included 24 immature rabbits and 24 mature rabbits. Tears were created in the anterior horn of medial meniscus of right knee in each rabbit. Animals were sacrificed at 1, 3, 6, and 12 weeks postoperatively. We performed macroscopic and histological evaluations of post-meniscal repair specimens. Cells were counted within a region of interest to confirm cellularization at tear site in immature menisci. The width of cell death zone was measured to determine the region of cell death in mature menisci. Apoptosis was evaluated by TUNEL assay. Vascularization was assessed by CD31 immunofluorescence. The glycosaminoglycans and the types 1 and 2 collagen content was evaluated by calculating average optical density of corresponding histological specimens. Cartilage degeneration was also evaluated. Healing outcomes following untreated meniscal tears were superior in immature group. Recellularization with meniscus-like cell morphology was observed at tear edge in immature menisci. Superior recellularization was observed at meniscal sites close to joint capsule than at sites distant from the capsule. Recellularization did not occur at tear site in mature group; however, we observed gradual enlargement of cell death zone. Apoptosis was presented at 1, 3, 6, 12 weeks in immature and mature menisci after untreated meniscal tears. Vascularization was investigated along the tear edges in immature menisci. Glycosaminoglycans and type 2 collagen deposition were negatively affected in immature menisci. We observed glycosaminoglycan degradation in mature menisci and cartilage degeneration, specifically in immature cartilage of the femoral condyle. In conclusion, compared with mature rabbits, immature rabbits showed more robust healing response after untreated meniscal tears. Vascularization contributed to the recellularization after meniscal tears in immature menisci. Meniscal injury fundamentally alters extracellular matrix deposition.

## Introduction

Menisci, the semilunar wedges of fibrocartilaginous tissue located between the femoral condyle and tibial plateau, play a key role in load bearing, load transmission, shock absorption, and lubrication during femorotibial articulation and dynamic knee movements (Newman et al., 1989; Zhu et al., 1994; Makris et al., 2011). An epidemiological study reported that meniscal injuries represented the most frequent knee joint-related injuries in the United States, and the incidence of meniscal injuries in the right knee ranged from 19% (95% confidence interval [CI] 15–24) among women aged 50–59 years to 56% (95% CI 46–66) among men aged 70–90 years (Englund et al., 2008). Another epidemiological investigation on the incidence of meniscal injuries among high school athletes during the 2007–2008 and 2012–2013 academic years showed an overall injury rate of 5.1/100000 athlete exposures (Mitchell et al., 2016). Therefore, evidently, meniscal injuries occur across a wide range of age groups that range from children, young adults, elderly individuals, as well as the general population, and athletes.

Age is a key factor that affects meniscal repair; increased age is associated with unfavorable outcomes (Kwon et al., 2019). A previous study that investigated the outcomes of arthroscopic meniscal repair in young patients over a mean follow-up of 27 months reported excellent postoperative clinical outcomes in 43 of 45 patients, regardless of the interval between injury and surgery or tear location (Vanderhave et al., 2011). A systematic review concluded that meniscal repair in children and adolescents was associated with good-to-excellent outcomes, regardless of the tear pattern, zone, or repair techniques (Ferrari et al., 2019). Overall, the repair outcomes of meniscal tears were better in younger than in older. However, to our knowledge, the exact mechanisms that contribute to the superior meniscal repair potential in younger are unclear from a histological perspective.

The lapine animal model is commonly used in comparative experimental studies, including in research on meniscal repair (Zhang et al., 2019a; Chen et al., 2020; Kim et al., 2021). The robustness of healing potential is better in this animal model than in models that use larger animals, such as pigs, goats (Ahern et al., 2009; Chu et al., 2010). Therefore, this model facilitates investigation of the spontaneous healing process after untreated meniscal tears and scores over other animal models in this field. This study investigated meniscal tears in rabbits at two different developmental stages (immature and mature). The first stage of the study included macroscopic and histological evaluation of the degree of spontaneous meniscal healing after untreated meniscal tears. The second stage involved histological evaluation using toluidine blue and Safranin O-fast green stains and immunohistochemical analysis (type 1 and 2 collagen) to determine changes in glycosaminoglycan (GAG) and collagen content of the extracellular matrix (ECM) after untreated meniscal tears. Finally, we performed macroscopic and histological evaluation of the degree of cartilage degeneration secondary to meniscal tears. Our hypotheses were as follows: 1) Compared with mature rabbits, immature rabbits showed more robust healing response of untreated meniscal tears. 2) Meniscal injury fundamentally alters ECM deposition.

## Materials and Methods

### Study Design and Surgical Procedure

**FIGURE 1:**
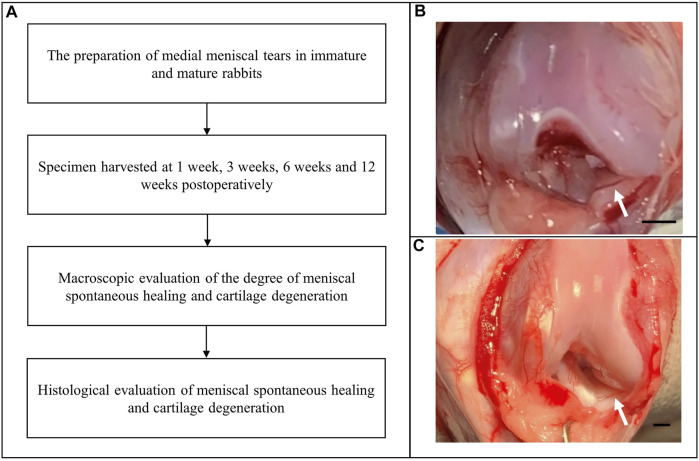
Flow diagram showing creation of the medial meniscal tear model. **(A)** The experimental flow diagram. **(B)** Creation of “parrot beak” tears in the anterior horn of the medial meniscus of the right knee in immature rabbits. Scale bar: 1 mm. **(C)** Creation of “parrot beak” tears in the anterior horn of the medial meniscus of the right knee in mature rabbits. The white arrows indicate meniscal tears. Scale bar: 1 mm.

This study was approved by the Institutional Laboratory Animal Ethics Committee, and all experimental procedures were performed in accordance with the National Institutes of Health Guide for the Care and Use of Laboratory Animals. The study included 24 immature female New Zealand white rabbits and 24 mature female rabbits. Clinically, the vertical “parrot beak” meniscal tear (a radial tear with an oblique course) is associated with an unstable partially torn meniscal flap and consequently poor healing capacity. This type of tear model can be easily prepared in research studies and does not require significant destruction of soft tissues to expose the meniscus. Therefore, we created vertical “parrot beak” meniscal tears in the anterior horn of the medial meniscus of the right knee, and the intact medial meniscus of the left knee was used as a control. Animals were sacrificed at 1, 3, 6, and 12 weeks postoperatively. Each group included six rabbits. We performed macroscopic and histological evaluations to determine the degree of spontaneous meniscal healing and cartilage degeneration (Figure 1A).

The animals were administered an intramuscular injection of xylazine hydrochloride (2 mg/kg) and placed in a supine position after general anesthesia. Following standard skin preparation and disinfection procedures, a medial parapatellar incision was made to dislocate the patella. The soft tissues adherent to the anterior horn of the medial meniscus were carefully pulled with forceps to enable complete exposure of the anterior horn. Using a pair of corneal scissors, we created “parrot beak” meniscal tears. Immature meniscal tears measured 1 mm in length with a rim width of 1 mm (Figure 1B). Mature meniscal tears in the poorly vascularized (“white-white”) zone measured 2.5 mm in length with a rim width of 1.5 mm (Figure 1C) to maintain the meniscal tears in the poorly vascularized zone. The meniscal tears remained untreated to simulate the tear instability usually observed in clinical practice. The incision was then closed using a continuous suture. The animals received a penicillin sodium injection to prevent infection and were allowed to move freely in a comfortable environment.

### Macroscopic and Histological Evaluation of Menisci

The entire medial meniscus was harvested from both the right and left knees and photographed in all rabbits, 1, 3, 6, and 12 weeks postoperatively. The menisci were fixed in 4% paraformaldehyde and embedded in paraffin after dehydration in graded ethanol solution. The specimens were cut in a transverse plane to include the anterior horn, the body, and the posterior horn. Subsequently, 4 µm-thick paraffin sections were stained using hematoxylin-eosin (HE), toluidine blue, Safranin O-Fast green, and immunohistochemical (IHC) stains for types 1 and 2 collagen and CD31. The IHC stains of types 1 and 2 collagen were used to evaluate the content of collagen within the meniscal ECM. The IHC staining of CD31 was used to detect vascularization. Details of the staining procedure are described by previous studies (Yan et al., 2020a; Yan et al., 2020c).

The meniscal scoring system described by Zellner et al. (Zellner et al., 2013) was used for semiquantitative assessment of healing outcomes of meniscal tears. This scoring system includes the macroscopic and histological features of repaired menisci. Macroscopic evaluation included evaluation of stability and filling of the defect. The following variables were included in histological evaluation: the surface area quality, integration, cellularity, cell morphology, and the proteoglycan and type 2 collagen content of the repaired tissue. Scores ranging from 0 to three were graded into eight individual subgroups. The final scores ranging from 0 points (no evidence of repair) to 24 points (complete repair) are presented in Supplementary Table S1.

### Semiquantitative Analysis of Histological Changes in Menisci

We counted the cells within a region of interest (ROI) in specimens stained with HE to determine recellularization in immature meniscal tears. Cell death was observed within a width of approximately 100 μm on either side of the tear edge at 1 W, postoperatively. Cell counting was performed within the ROI (200 μm in length, 100 μm in width) at sites close to and distant from the peripheral joint capsule to evaluate the degree of recellularization. We counted six menisci in each group except for the 3 and 12 W groups, because two menisci in the 3 W and one meniscus in the 12 W group healed completely. Mature menisci did not show evidence of recellularization following meniscal tears. The region of cell death referred to as the “dead zone” showed gradual enlargement over time. We also measured the width of the “dead zone” in mature menisci (*n* = 6) in this study. In this section, the slices were scanned by a digital slide scanner (NanoZoomer, Hamamatsu).

Using the ImageJ software (US National Institutes of Health), we calculated the average optical density (AOD) within the ROI (200 μm in length, 100 μm in width) to semiquantitatively evaluate the GAG content adjacent to the tear edge (*n* = 6). The GAG content within the same ROI at the corresponding area of the contralateral intact meniscus served as the control. Toluidine blue and Safranin O stains both reflect GAG content. In this study, the toluidine blue stain was used to measure GAG content, and the GAG content in Safranin O-Fast green stained samples was omitted from the analysis. The type 1 and 2 collagen content was measured using the same methods.

### Apoptosis Examination

Four slices of each spontaneous healing group were randomly selected for cell apoptosis evaluation within meniscal tissue by the TUNEL assay (DeadEnd™ Fluorometric TUNEL System, Promega, G3250). The detection process was performed according to the manufacturer’s instructions. Briefly, the 3 μm thick paraffin-embedded sections were immersed into fresh xylene and graded ethanol to deparaffinize and regain water. Then, after permeabilization with 20 ug/ml proteinase K solution and equilibration with equilibration buffer, the tissue sections were incubated with fluorescein-12-dUTP for 1 hour at 37°C. Then, add DAPI nuclear stain in mounting medium and proceed to analysis. The apoptotic cells were demonstrated with localized green fluorescence (fluorescein-12-dUTP) in a blue background (DAPI) when detected by confocal fluorescence microscope (TCS-SP8, Leica). The apoptosis ratio was defined by the following formula: number of cells stained by fluorescein-12-dUTP divided by number of cells stained by DAPI.

### Evaluation of Cartilage Degeneration

The macroscopic appearance of the medial femoral condyle (MFC) and medial tibial plateau (MTP) was assessed. Macroscopic changes in the anterior portion of the MFC and MTP were rated based on the Outerbridge classification (Outerbridge, 1961) (Supplementary Table S2), because the meniscus tear was created in the anterior horn, which is in close contact with the anterior portion of the MFC and the MTP. The anterior portion of the MFC and MTP was divided based on the procedure described by a previous study (Zhang et al., 2019b).

After fixation using 4% paraformaldehyde, the MFC and MTP were subjected to 5 days hydrochloric acid treatment for decalcification. The specimens were embedded in paraffin and cut into 3 μm sections. Histological specimens were stained using HE stain for morphological evaluation. The GAG content of cartilage was evaluated using toluidine blue. The sections were then scanned by a digital slide scanner (NanoZoomer, Hamamatsu), and cartilage degeneration was graded based on the osteoarthritis cartilage histopathology assessment (Osteoarthritis Research Society International [OARSI] system (Pritzker et al., 2006; Yan et al., 2020b) (Supplementary Table S3).

### Statistical Analysis

A priori power analysis using the G*Power software (G*Power, version 3.1.9.2) was used to calculate the appropriate sample size for this study. A sample size of six menisci in each group was necessary to achieve a power of 0.8, an effect size of 0.8, at the *α* level of 0.05. All data points are expressed as median values with 95% CIs. The one-way analysis of variance (ANOVA) with the Bonferroni multiple comparison test was used for comparison of the “dead zone” width in mature menisci. Before-after plots were used to compare between the tear (experimental) and control groups with regard to post-injury changes in meniscal ECM deposition, reflected by GAG and collagen one and two indices. The repeated measure two-way ANOVA along with the Bonferroni multiple comparison test was used for pairwise comparison of ECM deposition (GAG, collagen one and two content) between the tear and control groups. The ordinary two-way ANOVA with the Bonferroni multiple comparison test was used for other comparisons. All statistical analyses were performed using the GraphPad Prism software, version 8.0.1 (GraphPad Software). A *p* value <0 .05 was considered statistically significant.

## Results

### Macroscopic Evaluation and Meniscal Repair Scores

Meniscal tears in immature menisci showed no junction at the tear interface 1 W postoperatively. Macroscopic evaluation performed 3 W postoperatively showed completely healed menisci in specimens NO. 1 and 3R. However, other specimens showed inferior meniscal repair with an apparent gap. Macroscopic evaluation at 6 W postoperatively showed a junction at the tear interface in all specimens, except for a distinct gap in specimen NO. 2R. Macroscopic evaluation performed at 12 W postoperatively showed a connection at the tear interface in all specimens, except for a distinct gap in specimen NO. 6R. The synovial membrane was attached to the tear edge in all specimens. A distinct gap was present in nearly all mature menisci at 1, 3, 6, and 12 weeks postoperatively, despite a weak connection observed at 6 weeks in some specimens, such as specimens NO. 3 and 6R. However, no specimen could withstand the pulling stress under traction using forceps at the tear site, showing unstable tear edges (Supplementary Figure S1A).

The overall meniscal scores were better in the immature than in the mature group. We observed significant differences between the immature and mature groups at 3 and 12 W postoperatively (*p* < 0.01). Meniscal scores recorded 6 W postoperatively were higher in the immature than in the mature group, although the difference was statistically nonsignificant (*p* > 0.05). The meniscal score in the immature group increased with extension of investigation time (immature group 1 vs 3 W, *p* < 0.0005, immature group 1 vs 12 W, *p* < 0.0005). However, meniscal scores in the mature group were lower throughout the entire study period (Supplementary Figure S1, b).

### Cellular Changes and Vascularization in Immature and Mature Menisci

**FIGURE 2:**
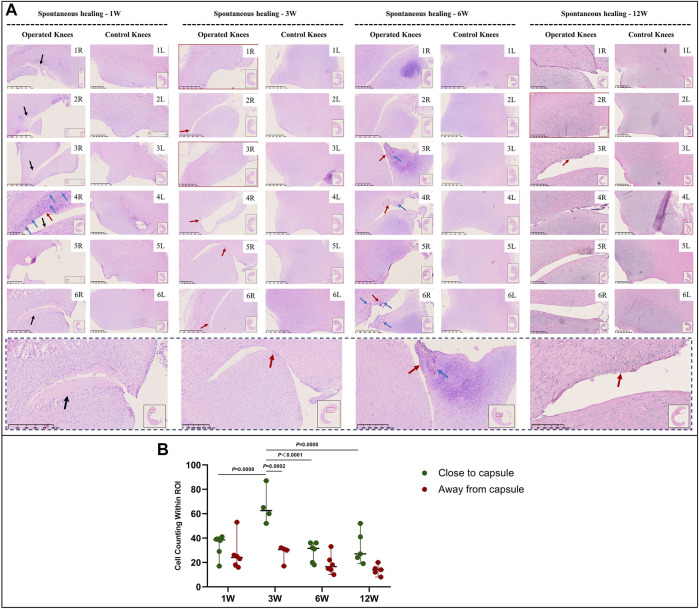
HE staining and recellularization evaluation in immature menisci. **(A)** HE staining. **(B)** Cell counting within the ROI for evaluation of recellularization close to and distant from the joint capsule in immature menisci. The serial number in the upper right corner of each macroscopic image indicates the number of each specimen. The black arrows indicate the area of cell death. The red arrows indicate cell clusters. The blue arrows indicate blood vessels. Images with a red border indicate histological specimens showing completely healed menisci. The lower panel of images with a black dotted-line border indicate representative specimens in the corresponding observation period. The cell counts within the ROI are expressed as median values with 95% CIs. The sample size for each group was six except for the 3 W (*n* = 4) and the 12 W group (*n* = 5); this discrepancy was owing to complete healing observed in two samples at 3 W and one sample at 12 W. W: weeks.

In the immature group, cell death was observed within a width of approximately 100 μm on either side of the tear edge, 1 W postoperatively. Cell clusters and blood vessels were observed in specimen NO. 4R. No cellularity was observed in any other specimen. Recellularization was observed in all samples 3 W postoperatively, with complete healing in two samples (NO. 1 and 3R). Persistent recellularization and cell clusters were observed at 6 and 12 weeks postoperatively (Figure 2A). The number of cells was significantly higher at tear sites close to the joint capsule than at sites distant from the capsule, 3 W postoperatively (*p* < 0.0005), although no significant differences were observed at 1, 6, and 12 W (Figure 2B). Recellularization at the tear edge close to the joint capsule was maximal at 3 W compared with recellularization observed at 1, 6, and 12 W. The cellular apoptosis was presented at 1,3,6,12W (Figure 4). The typical characteristic phenomenon of pyknosis (condensation of chromatin) at the early stage of apoptosis emerged (Supplementary Figure S2, a). The vascularization demonstrating CD31 positive was observed along the tear edges in immature group throughout the whole experimental period (Figure 5).

**FIGURE 3:**
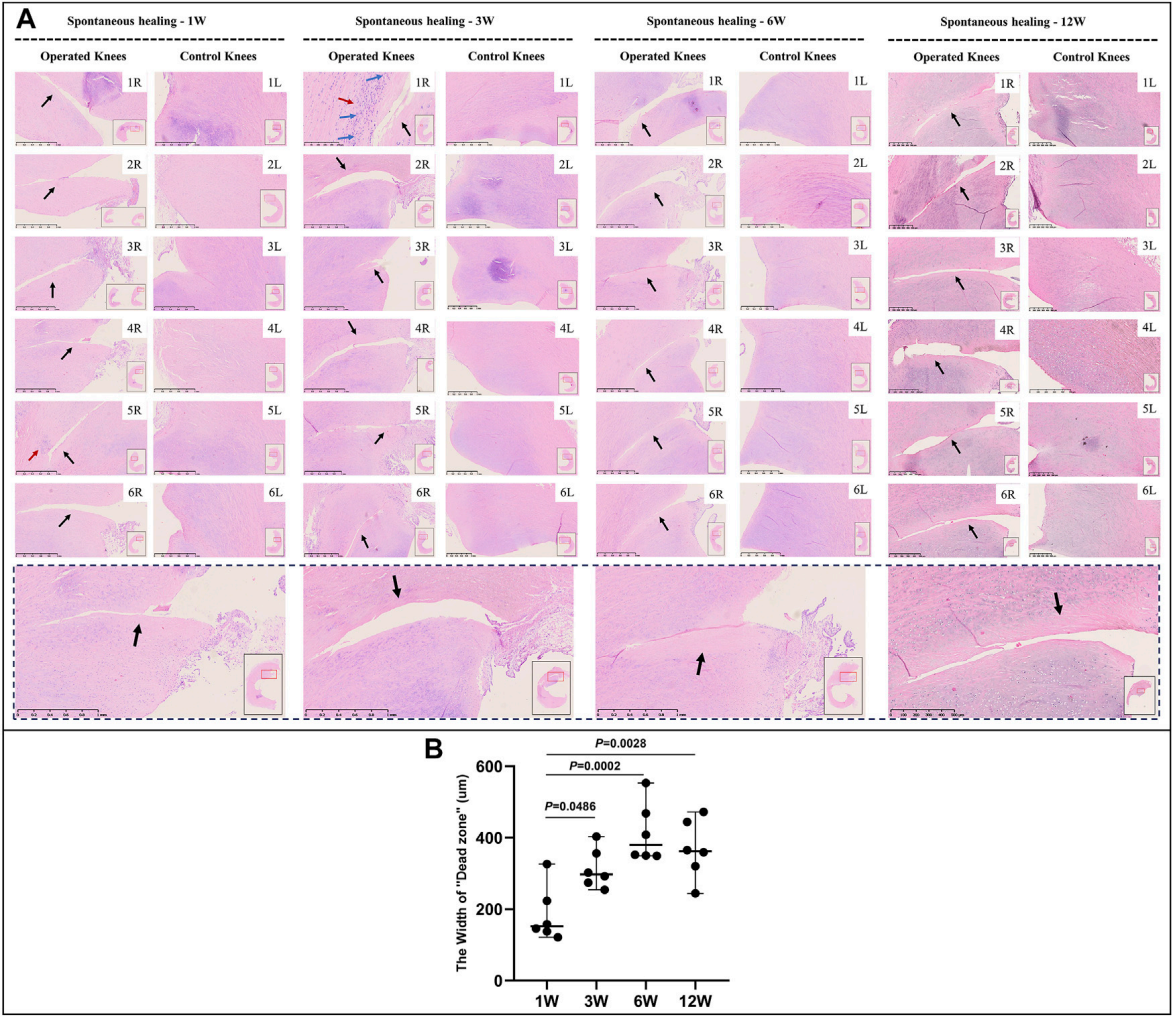
HE staining and evaluation of cell death in mature menisci. **(A)** HE staining. **(B)** Evaluation of the width of the “dead zone” in torn mature menisci. The black arrows indicate the area of cell death (“dead zone”). The red arrows indicate cell clusters. The blue arrows indicate blood vessels. The lower panel of images with a black dotted-line border indicate representative specimens in the corresponding observation period. The width of the “dead zone” is expressed as median values with 95% CIs. The sample size for each group is six.

**FIGURE 4:**
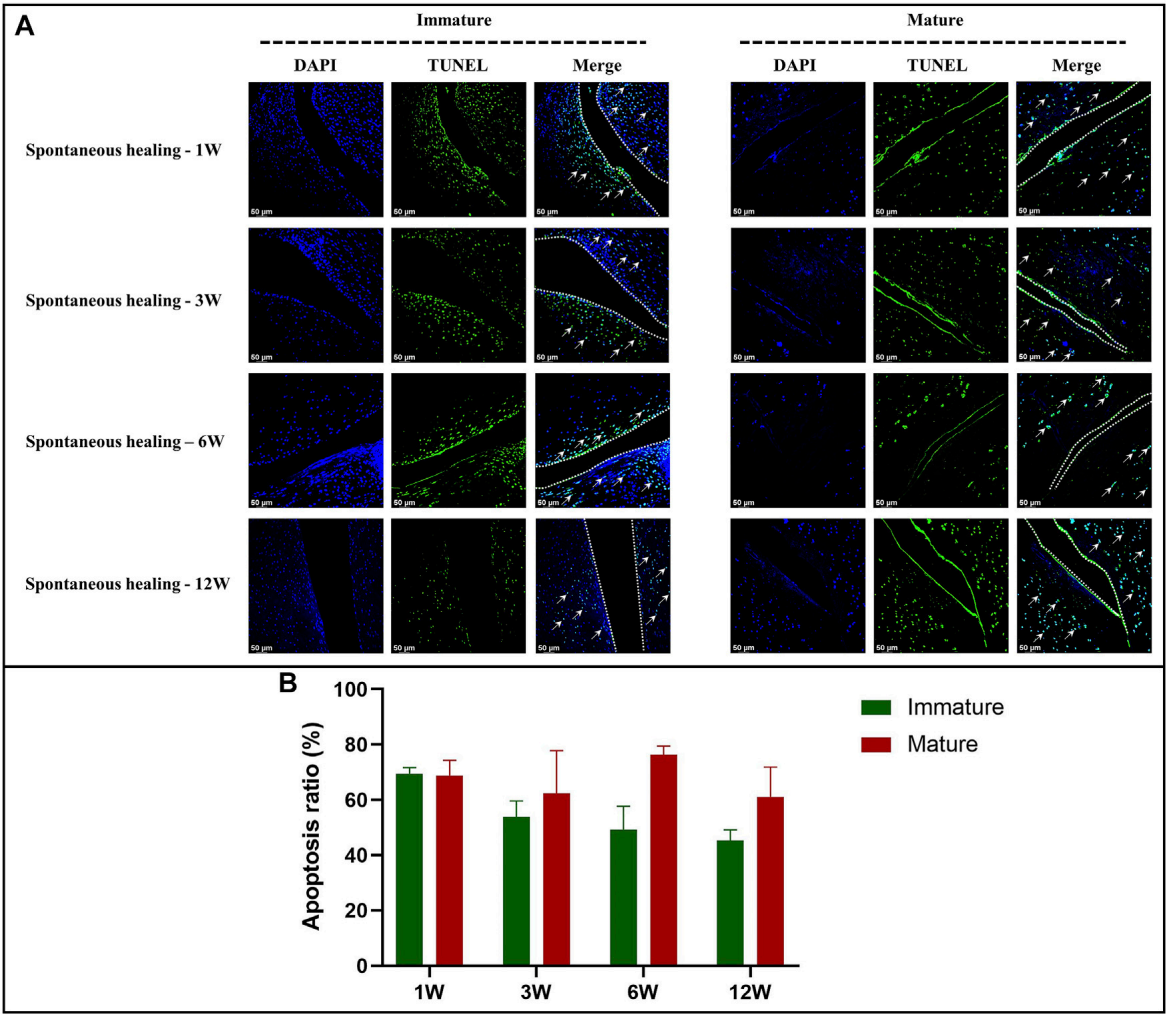
The assessment of apoptosis in immature and mature menisci after untreated meniscal tears. **(A)** TUNEL staining. The white arrows indicate apoptotic cells. The dotted white lines indicate tear edges. **(B)** Apoptosis ratio. The values are expressed as median values with 95% CIs. *n* = 4.

**FIGURE 5:**
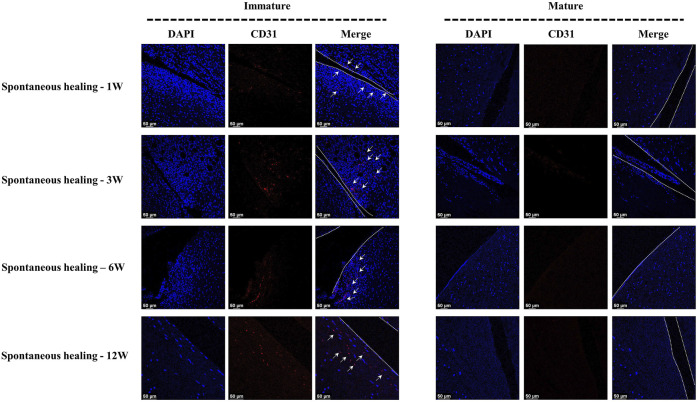
The assessment of vascularization in immature and mature menisci after untreated meniscal tears. The white arrows indicate CD31 positive cells. The dotted white lines indicate tear edges.

Mature menisci showed cell death despite regional cell aggregation, such as observed in specimen NO. 5R, 1 W postoperatively. Regional cell aggregation and newly formed blood vessels were identified only in specimen NO. 1R, 3 W postoperatively. The “dead zone” was observed at 1, 3, 6, and 12 W and showed gradual overall enlargement. No recellularization occurred in the mature group throughout the study period (Figure 3A). The overall width of the “dead zone” showed gradual enlargement (Figure 3B) and was maximal at 6 W compared with the width at 1, 3, and 12 W (mean width 413 μm at 6 W vs 185 μm at 1 W, 313 μm at 3 W and 367 μm at 12 W). The cellular apoptosis was presented at 1,3,6,12 W (Figure 4). The typical characteristic phenomenon of pyknosis (condensation of chromatin) at the early stage of apoptosis emerged (Supplementary Figure S2, B). For mature group, no obvious vascularization with CD31 positive expression was investigated along the tear edges (Figure 5).

### Glycosaminoglycan Deposition Following Meniscal Tears

**FIGURE 6:**
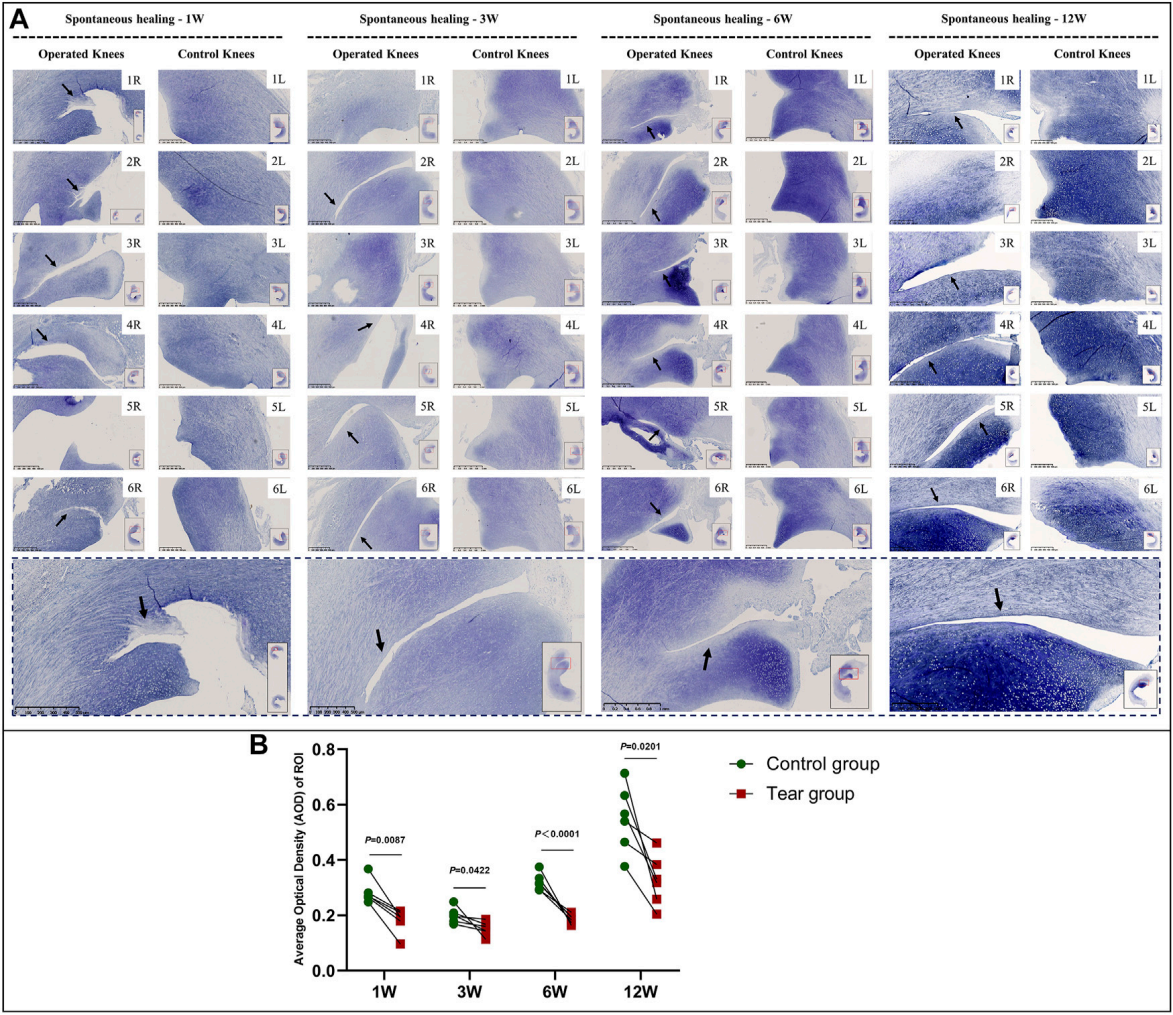
Toluidine blue staining and semiquantitative evaluation of GAG content in immature menisci. **(A)** Toluidine blue staining. **(B)** Semiquantitative evaluation of GAG content. The black arrows indicate fading of toluidine blue stain. The lower panel of images with a black dotted-line border indicate representative specimens in the corresponding observation period. AOD values are expressed as median values with 95% CIs. *n* = 6.

The overall GAG content tended to increase with meniscal development in the immature group. GAG deposition at the tear site was lesser to that at the corresponding area of the contralateral native menisci, except in specimens NO. 1 and 3R, which showed complete healing at 3 W and in specimen NO. 2R, which was evaluated at 12 W. The reduction area of GAG deposition showed gradual enlargement without recovery at the time of final follow-up (Figure 6, a and Supplementary Figure S3). Semiquantitative analysis results were consistent with those obtained using toluidine blue stain (Figure 6B). Significant differences were observed between the tear and control groups at 1 W (mean average optical density [AOD] 0.18 vs. 0.28, *p* < 0.01), 3 W (mean AOD 0.15 vs. 0.20, *p* < 0.05), 6 W (mean AOD 0.19 vs 0.32, *p* < 0.0001) and 12 W (mean AOD 0.33 vs. 0.55, *p* < 0.05), postoperatively. The greatest difference in GAG content between the tear and control groups was observed at 12 W.

**FIGURE 7:**
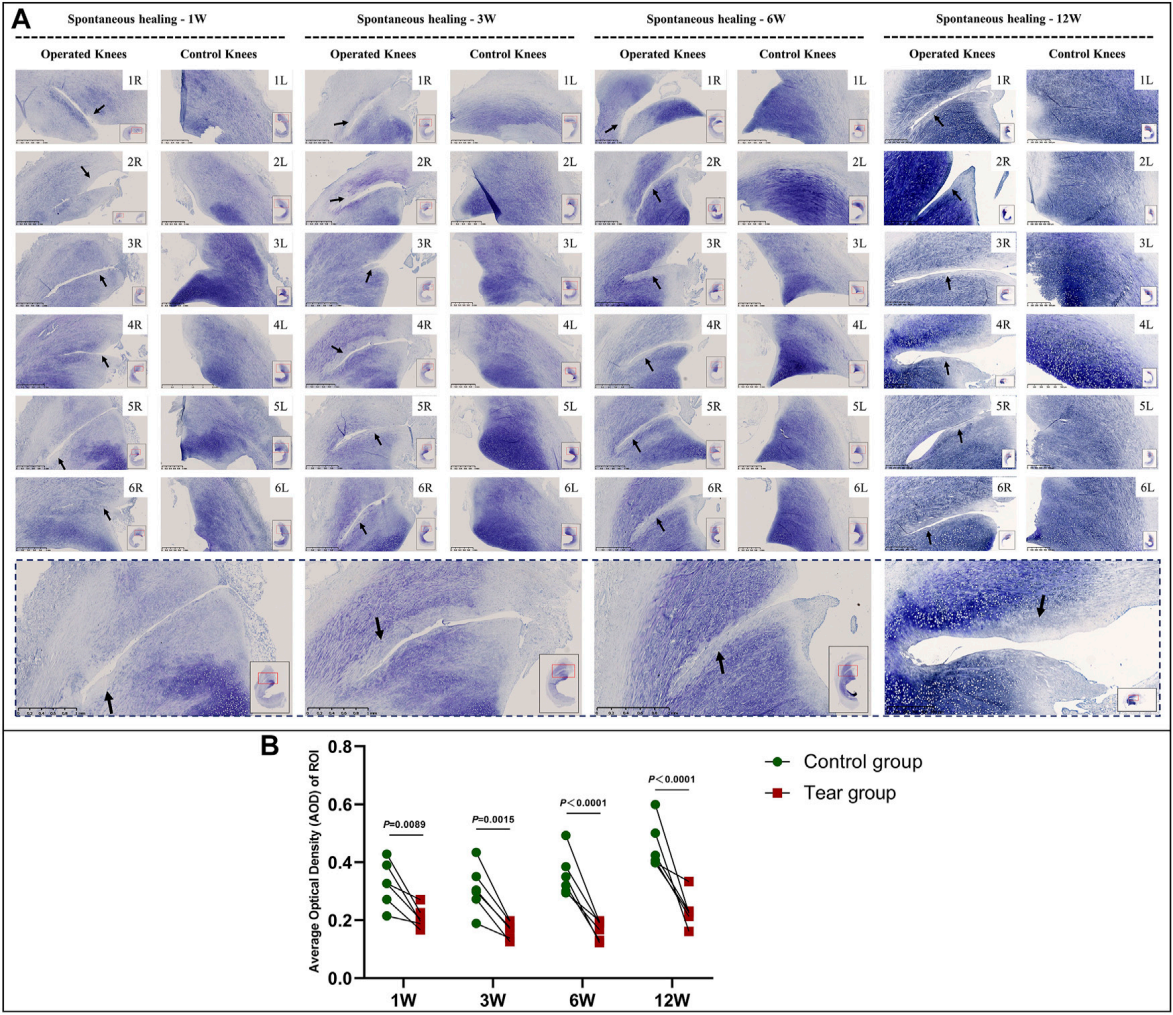
Toluidine blue staining and semiquantitative evaluation of GAG content in mature menisci. **(A)** Toluidine blue staining. **(B)** Semiquantitative evaluation of GAG content. The black arrows indicate the fading of toluidine blue stain. The lower panel of images with a black dotted-line border indicate representative specimens in the corresponding observation period. AOD values are expressed as median values with 95% CIs. *n* = 6.

Reduction in the GAG content at the tear site was observed in all specimens in the mature group. The reduction area of GAG deposition showed gradual enlargement without recovery at the final follow-up. Moreover, the reduction area of GAG corresponded to the aforementioned “dead zone” observed in mature menisci (Figure 7, a and Supplementary Figure S4). The semiquantitative analysis results were consistent with the aforementioned findings; we observed significant differences in GAG content between the tear and control groups, 1 W (mean AOD 0.21 vs 0.33, *p* < 0.01), 3 W (mean AOD 0.17 vs 0.31, *p* < 0.01), 6 W (mean AOD 0.17 vs 0.36, *p* < 0.0001), and 12 W (mean AOD 0.23 vs 0.45, *p* < 0.0001) postoperatively (Figure 7B).

### Type 1 and 2 Collagen Deposition Following Meniscal Tears

**FIGURE 8:**
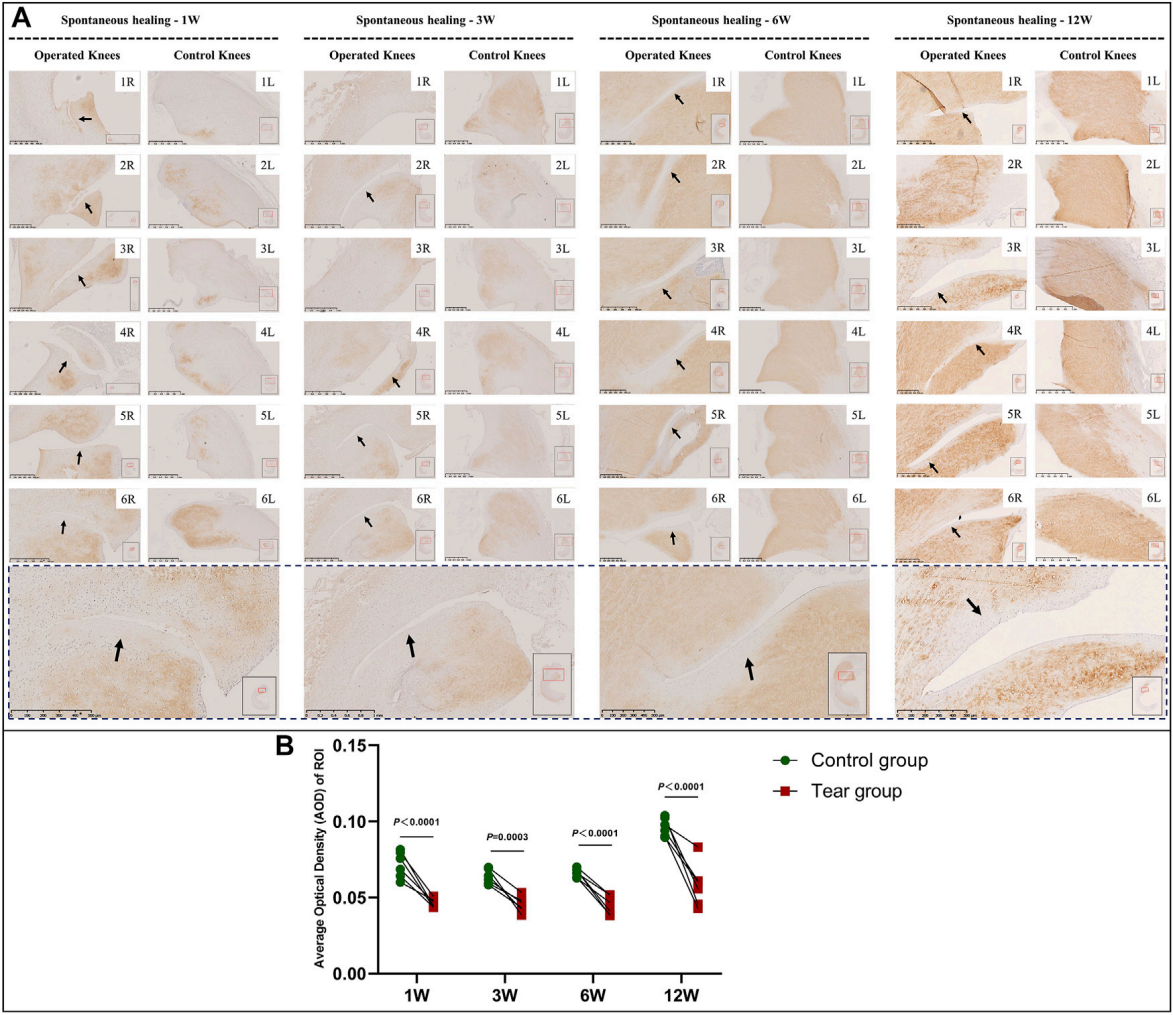
Immunohistochemical and semiquantitative evaluation of type 2 collagen content in immature menisci. **(A)** Immunohistochemical staining of type 2 collagen. **(B)** Semiquantitative evaluation of type 2 collagen content. The black arrows indicate fading of diaminobenzidine (DAB) stain used for type 2 collagen. The lower panel of images with a black dotted-line border indicate representative specimens in the corresponding observation period. AOD values are expressed as median values with 95% CIs. *n* = 6.

**FIGURE 9:**
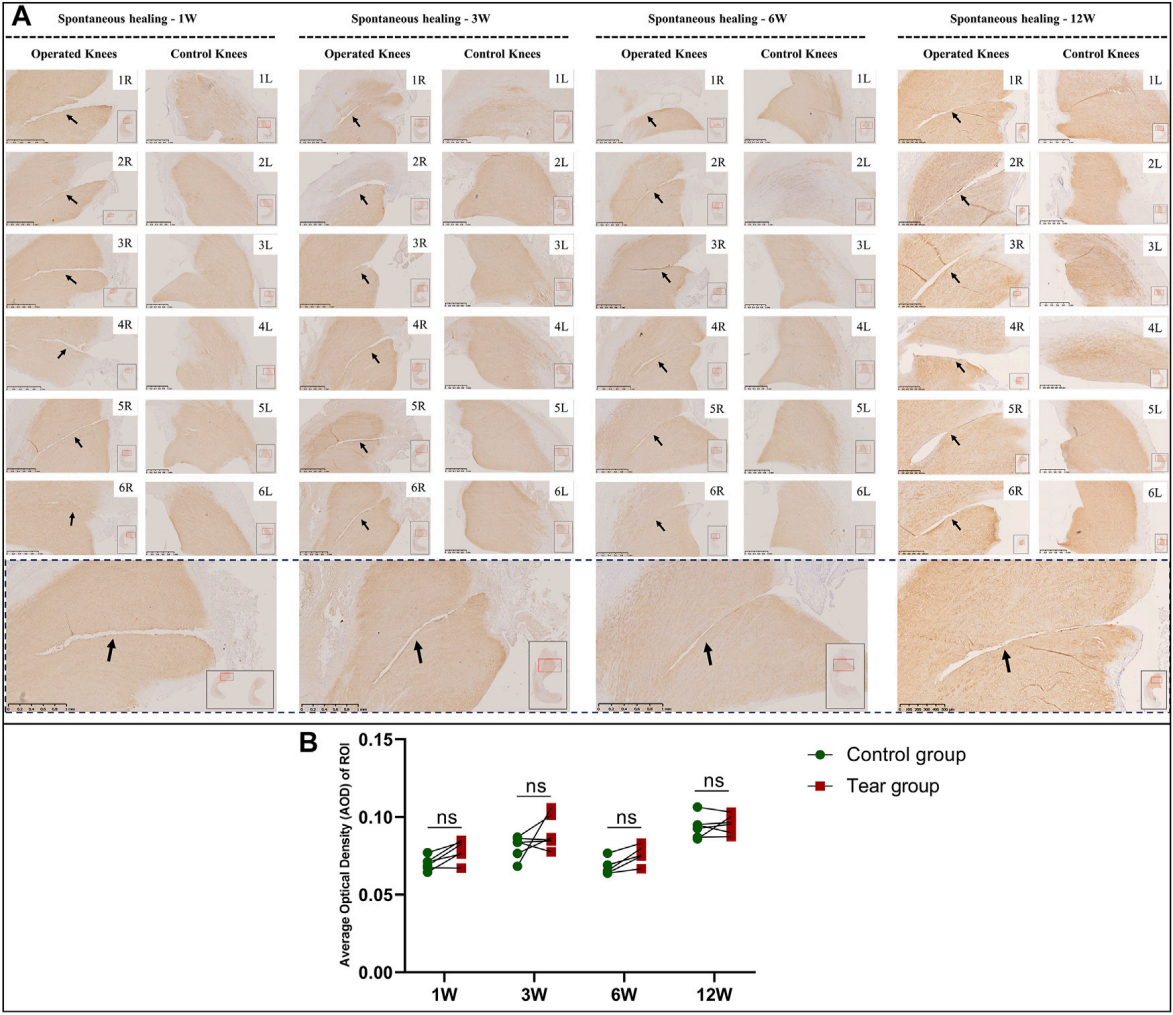
Immunohistochemical and semiquantitative evaluation of type 2 collagen content in mature menisci. **(A)** Immunohistochemical staining of type 2 collagen. **(B)** Semiquantitative evaluation of type 2 collagen content. The black arrows indicate the tear site. The lower panel of images with a black dotted-line border indicate representative specimens in the corresponding observation period. AOD values are expressed as median values with 95% CIs. *n* = 6.

In the immature group, the overall type 2 collagen content gradually increased with meniscal development. Type 2 collagen deposition at the tear edge was lesser to that observed at the corresponding region of the contralateral native meniscus, except for specimens NO. 1 and 3R, which showed complete healing at 3 W and specimen NO. 2R, which was completely healed at 12 W. No recovery of type 2 collagen deposition was observed at the tear site at the final follow-up (Figure 8A). Semiquantitative analysis results were consistent with those of type 2 collagen staining (Figure 8B). Significant differences were observed between the tear and control groups, 1 W (mean AOD 0.05 vs 0.07, *p* < 0.0001), 3 W (mean AOD 0.05 vs 0.06, *p* < 0.0005), 6 W (mean AOD 0.04 vs 0.07, *p* < 0.0001), and 12 W (mean AOD 0.06 vs 0.10, *p* < 0.0001) postoperatively. In the mature group, type 2 collagen deposition at the tear edge was comparable with that observed at the corresponding region of the contralateral native meniscus (Figure 9A). No significant differences were observed in type 2 collagen content between the tear and control groups at 1, 3, 6, and 12 W (Figure 9B). However, the content of type 2 collagen in mature menisci was superior to that of immature menisci at each time point after untreated meniscal tears (Supplementary Figure S5).

In the immature group, abundant type 1 collagen was deposited throughout the meniscus. The deposition of type 1 collagen at the tear site was comparable with that deposited at other sites, regardless of the area (Supplementary Figure S6, a). No significant differences were observed in the AOD of type 1 collagen between the tear and control groups at 1, 6, and 12W (Supplementary Figure S6, b), except for 3 W (mean AOD 0.12 vs 0.13, *p* < 0.05). Similar results were observed in mature menisci (Supplementary Figure S7, a). However, no significant differences were observed in the AOD of type 1 collagen between the tear and control groups at 1, 3, 6, and 12W (Supplementary Figure S7, b).

### Cartilage Degeneration Following Meniscal Tears

**FIGURE 10:**
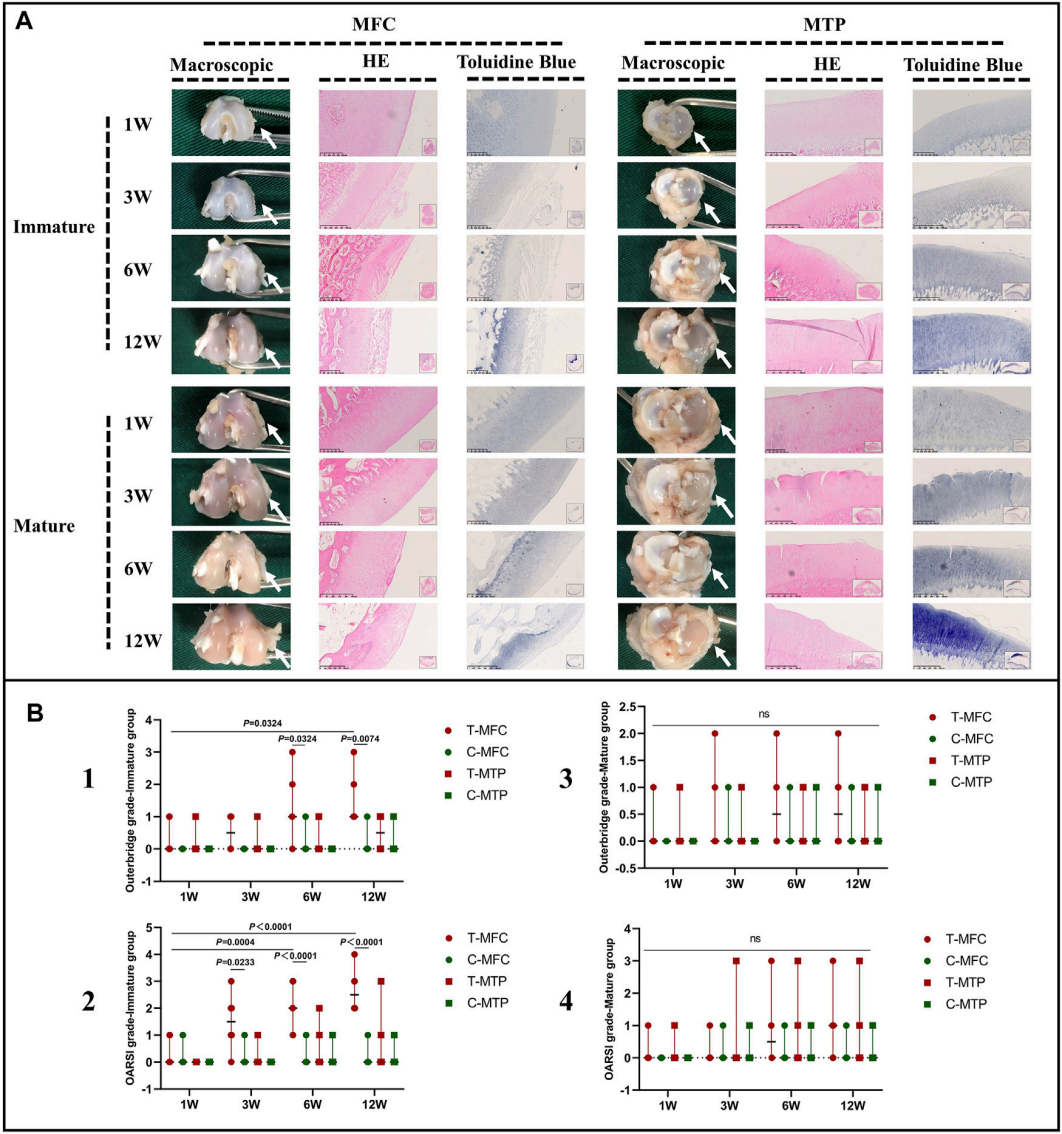
Macroscopic and histological evaluation of cartilage degeneration. **(A)** Macroscopic and histological evaluation of MFC and MTP cartilage degeneration in the immature and mature groups. **(B)** Outerbridge scoring and histological OARSI scoring for cartilage degeneration. The white arrows indicate the MFC or MTP. Scores are expressed as median values with 95% CIs. *n* = 6. ns: no significant differences.

In the immature group, macroscopic evaluation showed a smooth surface of the MFC and MTP, 1 W postoperatively. Histological evaluation showed similar results with morphologically intact cartilage and normal matrix architecture. A mild uneven surface and toluidine blue depletion were observed on histological evaluation, 3 W postoperatively. Macroscopic and histological evaluation showed worsened cartilage degeneration, 6 and 12 W postoperatively. Macroscopically, we detected an uneven surface and abrasions in the MFC. Histological evaluation of HE stained specimens showed superficial cartilaginous fibrillation, cartilage erosions, and prominent cell death in the MFC. Toluidine blue depletion in the MFC indicated reduced GAG deposition. However, the aforementioned findings were not observed in the MTP (Figure 10A). Outerbridge and OARSI scores were consistent with the aforementioned findings in the immature group (Figure 10, b1 and b2).

In the mature group, macroscopic evaluation of the MFC and MTP showed an even surface without cartilage abrasions 1, 3, and 6 W postoperatively, and histological evaluation showed findings consistent with those of macroscopic evaluation. Mild cartilage abrasions were observed in the MFC, 12 W postoperatively. A mild uneven surface and toluidine blue stain depletion were observed on histological evaluation. However, no clearly visible cartilage abrasions or degeneration was observed in the MTP on macroscopic or histological evaluation, even 12 W postoperatively. Outerbridge and OARSI scores were consistent with the aforementioned findings in the mature group (Figure 10, b3 and b4).

C-MFC: the medial femoral condyle in the control group, C-MTP: the medial tibial plateau of the control group, OARSI: Osteoarthritis Research Society International system, T-MFC: the medial femoral condyle in the tear group, T-MTP: the medial tibial plateau in the tear group.

## Discussion

This study highlights that overall spontaneous healing response of untreated vertical meniscal tears in the avascular zone was more robust in immature than in mature menisci. Interestingly, recellularization with meniscus-like cell morphology was observed at 3, 6, and 12 weeks postoperatively in immature menisci, despite cell death within an area that measured approximately 100 μm in width at the first week postoperatively. No recellularization occurred in mature menisci and peculiarly, the zone of cell death showed gradual enlargement. The presence of apoptosis was confirmed in immature and mature menisci after untreated meniscal tears at 1,3,6,12 weeks postoperatively. The initial GAG and type 2 collagen deposition were affected by tears of immature menisci. GAG in the ECM showed gradual post-tear degradation in mature menisci. Compared to mature menisci, immature menisci showed earlier and more significant post-tear cartilage degeneration, primarily in the cartilage of the femoral condyle.

Meniscal cell death was observed in both immature and mature menisci, 1 W postoperatively; similar findings are reported by previous studies (Uysal et al., 2008; López-Franco et al., 2011; Killian et al., 2014). Apparent cell death is known to occur in traumatic or degenerative tears of the human meniscus (Uysal et al., 2008). Studies have reported a significant acute reduction in cell viability 24 h post-meniscal tears (Killian et al., 2014). The present study confirmed the presence of apoptosis after meniscal injuries, which was consistent with previous studies (Hashimoto et al., 1999; Killian et al., 2014). The exact mechanisms underlying cell apoptosis after meniscal injury remain unclear. However, abnormal biomechanical stress that acts on meniscal tissue may serve as the primary trigger for meniscal cell apoptosis (Hashimoto et al., 1999). Persistent instability of the torn meniscus after tear creation in this study confirms the role of abnormal biomechanical stress. A previous study has reported that meniscal tear preparation disrupts local stress distribution and results in higher concentration of stress and longer and more unstable tears (Kedgley et al., 2019). Notably, synovial fluid analysis showed elevated levels of inflammatory mediators, including matrix metalloproteinase (MMP) and prostaglandin E2 after meniscal tears (Liu et al., 2017). Additionally, traumatic tears were followed by upregulation of MMP1 and MMP3 expression in the injured menisci (Brophy et al., 2017). Meniscal cell death may also be attributable to elevated expression of inflammatory mediators in the synovial fluid and meniscus, which necessitate prompt intervention to restore normal biomechanics, and administration of anti-inflammatory medication may prevent cell death after acute meniscal tears.

Interestingly, this study showed recellularization in immature menisci at 3,6,12 W postoperatively, which may be attributed to the specificity of the physiological characterizations of immature menisci. Firstly, previous studies have reported that the entire meniscus is fully vascularized from the time of prenatal development until shortly after birth, and only the peripheral 10–25% of the meniscal area is vascularized at maturity (Clark and Ogden, 1983; Makris et al., 2011). Adequate blood supply is essential for meniscal repair, which is reflected by the superior healing capacity of the peripheral vascularized zone (Makris et al., 2011; Kwon et al., 2019). Moreover, we also observed abundant vascularization along the tear edges after untreated meniscal tears in immature group. However, no distinct vascularization was investigated along the tear edges in mature group. Thus, vascularization contributed to the recellularization after untreated meniscal tears in immature individuals. Secondly, meniscus is populated by several different cell types with distinct phenotypes and cell membrane markers (Verdonk et al., 2005). One previous study characterized and compared meniscal cell changes in postnatal rat menisci at different developmental stages and showed that the number of cells decreased with maturity, with the highest cell density observed during the early postnatal phase. Moreover, meniscal cells observed during the early postnatal phase showed stem cell characteristics with high self-renewal capacity, cell proliferation, and differentiation potential (He et al., 2019). Thirdly, menisci are known to undergo structural transformation and alterations in the ECM composition with maturity. Compared with these features in immature menisci, mature menisci show a well-organized and dense ECM, with greater GAG and type 2 collagen deposition and organized collagen fiber arrangement (Melrose et al., 2005; Di Giancamillo et al., 2014; Gamer et al., 2017; Qu et al., 2018). A study has reported that the densely organized ECM in mature menisci impairs migration of meniscal cells secondary to the biophysical barrier presented by mature ECM; however, the ECM in immature menisci or partially enzymatically digested mature menisci produces the opposite effects (Qu et al., 2018). Therefore, rich blood supply, high cell densities and proliferative capacities and relatively loose ECM observed in immature menisci are considered initiators of recellularization. In contrast, recellularization did not occur in mature menisci and the “dead zone” showed a gradual increase in size in our study. Compared with immature menisci, mature menisci show poor vascularity, lesser cell densities, and densely organized ECM, all of which potentially interfere with recellularization. The differences between immature and mature menisci with regard to healing potential may be attributable to the discrepancies in recellularization and may contribute to the discrepancies in initiation of meniscal repair. Therefore, based on our study results, we propose that meniscal repair should be attempted for immature menisci, which is consistent with the current guidelines recommended for the management of meniscal tears in skeletally immature patients (Vanderhave et al., 2011; Ferrari et al., 2019).

In this study, we observed that GAG and type 2 collagen deposition was affected by meniscal tears in immature menisci. The cellular components that maintain and remodel meniscal tissue were deficient during the early post-tear phase in immature menisci, which was shown to negatively affect the deposition of ECM constituents. Deposition of GAG and type 2 collagen did not occur despite recellularization at the tear site in immature menisci. Similarly, GAG degradation was observed in mature menisci. The following mechanisms secondary to cell death may at least partially contribute to the destructive changes in the meniscal ECM: 1) The depletion of cellularity impairs the intrinsic capacity to maintain and remodel the meniscal tissue. 2) Cell apoptosis may cause ECM degradation (Hashimoto et al., 1998). Previous studies have shown that the turnover of proteoglycans (a matrix constituent) is faster than that of collagen (Matyas et al., 1999; Allen et al., 2006), which may potentially account for the greater GAG degradation compared with abundant residual type 1 and 2 collagen in mature menisci.

Studies have reported tear-induced cartilage degeneration (Dong et al., 2014; Killian et al., 2014; Zhang et al., 2019a; Li et al., 2020), which is consistent with the phenomenon of cartilage degeneration observed in our study, particularly in the immature cartilage of the femoral condyle. This finding may be attributed to the fact that the cartilage is subjected to abnormal biomechanics following meniscal tears (Zhang et al., 2019b). Moreover, the paradoxical movement between the femoral condyle and meniscus during joint motion may potentially account for the greater cartilage degeneration observed in the femoral condyle compared with the tibial plateau. Therefore, based on the findings of this study, we recommend that prompt intervention is important, particularly for immature meniscal tears, owing to the early and greater cartilage degeneration in this type of cartilage.

Following are the limitations of this study: 1) We only created a pure meniscal tear model in the anterior horn of the meniscus without any treatment administered to the animals; Further work is needed to evaluate the histology and immunohistochemistry after repair of meniscal tears in immature and mature rabbits to see if the cellularity is different once mechanical stability has been restored with a repair. 2) This study included only a 12 weeks observation period, without any further investigations of histological changes that might occur beyond 12 weeks. 3) The exact pathogenetic contributors to cell apoptosis remain unknown. Future studies should also investigate whether apoptosis could be reverted after meniscal reduction. 4) The present study used a rabbit model instead of a large animal model (for example, a porcine or goat model) in which physiological features more closely resemble those of humans.

## Data Availability

The original contributions presented in the study are included in the article/Supplementary Material further inquiries can be directed to the corresponding authors.
