# Supplementary material for: Histologically Confirmed Recellularization is a Key Factor that Affects Meniscal Healing in Immature and Mature Meniscal Tears
Source: Front Cell Dev Biol. 2021 Dec 8;9:793820. doi: 10.3389/fcell.2021.793820 (PMC8692889; doi:10.3389/fcell.2021.793820)
Supplement: Supplementary file 1 [file DataSheet1.docx]

**Supplemental files**

**Table S1:** Scoring System for Evaluation of the Quality of Meniscal Repair Tissue^a^

|  | 0 | 1 | 2 | 3 |
| --- | --- | --- | --- | --- |
| Defect filling | No fill | <25% | 25–75% | >75% |
| Surface | No surface | ruptured | Fissured/fibrillated | Meniscus-like |
| Integration | No integration | Partial, unilateral  integration | Bilateral partial or unilateral complete integration | Bilateral complete  integration |
| Cellularity | No cells | >10 cell cluster/slide | No cell cluster/slide,  Cell-ECM-ratio> 0.5 | Meniscus-like cell-ECM ratio |
| Cell morphology | No cells | <25% meniscus-like cells | 25%-75% meniscus-like cells | >75% meniscus-like cells |
| Content of  proteoglycan | No staining for  proteoglycan | <25% | 25–75% | >75% |
| Content of  type 2 collagen | No staining for  type 2 collagen | <25% | 25–75% | >75% |
| Stability | No stability | weak | Stable in shape | Stable to pressure and  pulling stress |

^a^ECM, extracellular matrix.

**Table S2.** Outerbridge classification

| Grade | Pathology |
| --- | --- |
| I | Softening and swelling of articular cartilage |
| II | Fragmentation and fissuring of articular cartilage affecting an area of less than 0.5 inches |
| III | Fragmentation and fissuring of articular cartilage affecting an area greater than 0.5 inches |
| IV | Cartilage erosion to bone |

**Table S3.** OA cartilage histopathology grade assessment

| Grade (key feature) | Associated criteria (tissue reaction) |
| --- | --- |
| Grade 0: surface intact, cartilage morphology intact | Matrix: normal architecture  Cells: intact, appropriate orientation |
| Grade 1: surface intact | Matrix: superficial zone intact, edema and/or fibrillation (abrasion), focal superficial matrix condensation  Cells: death, proliferation (clusters), hypertrophy, superficial zone reaction must be more than superficial fibrillation only |
| Grade 2: surface discontinuity | As above  + Matrix discontinuity at superficial zone (deep fibrillation)  ± Cationic stain matrix depletion (Safranin O or Toluidine Blue) upper 1/3 of cartilage  ± Focal perichondronal increased stain (mid zone)  ± Disorientation of chondron columns  Cells: death, proliferation (clusters), hypertrophy |
| Grade 3: vertical fissures | As above  Matrix vertical fissures into mid zone, branched fissures  ± Cationic stain depletion (Safranin O or Toluidine Blue) into lower 2/3 of cartilage (deep zone)  ± New collagen formation (polarized light microscopy, Picro Sirius Red stain)  Cells: death, regeneration (clusters), hypertrophy, cartilage domains adjacent to fissures |
| Grade 4: erosion | Cartilage matrix loss: delamination of superficial layer, mid layer cyst formation  Excavation: matrix loss superficial layer and mid zone |
| Grade 5: denudation | Surface: sclerotic bone or reparative tissue including fibrocartilage within denuded surface. Microfracture with repair limited to bone surface |
| Grade 6: deformation | Bone remodeling (more than osteophyte formation only). Includes: microfracture with fibrocartilaginous and osseous repair extending above the previous surface |


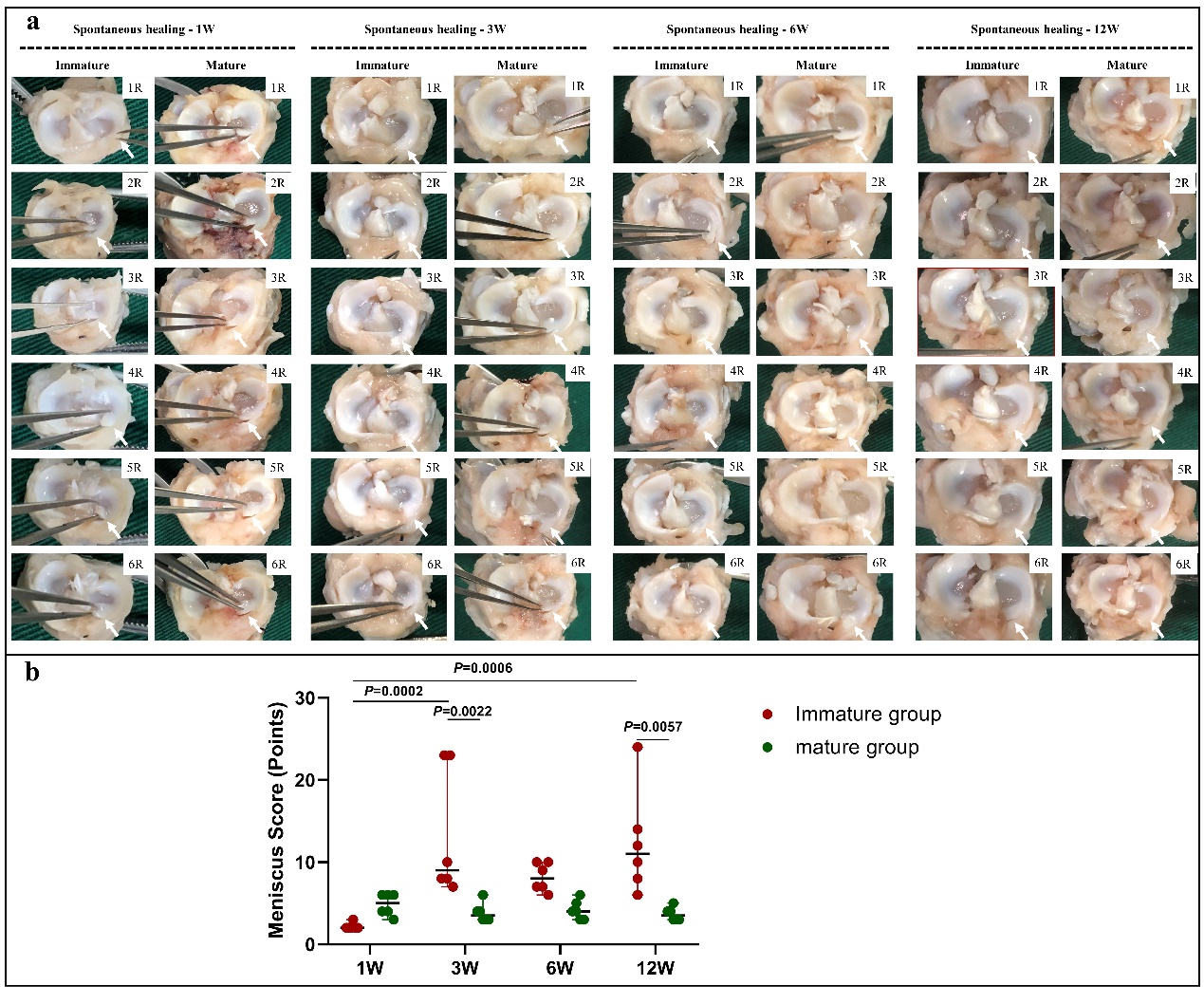


**Figure S1.** Macroscopic evaluation and the meniscal repair scoring system for spontaneous repair of immature and mature menisci. (a) Gross appearance of spontaneous healing of the entire meniscus. (b) The meniscal score for meniscus repair. The serial number in the upper right corner of each macroscopic image indicates the number of each specimen. The white arrow indicates the position (site) of the meniscus tear. The meniscal repair scores are expressed as median values with 95% CIs. n=6.


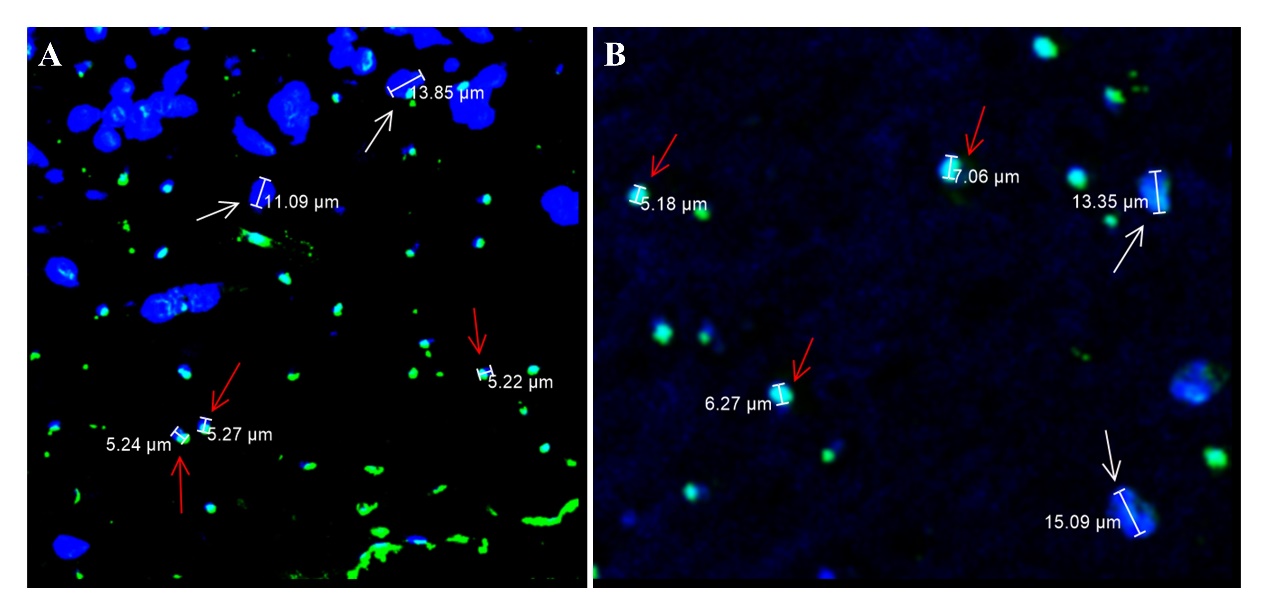


**Figure S2.** The presence of pyknosis (condensation of chromatin) in immature and mature menisci after untreated meniscal tears. A, pyknosis in immature menisci; B, pyknosis in mature menisci. The red arrows indicate pyknosis with reduced size of nucleus. The white arrows indicate relative normal nucleus.


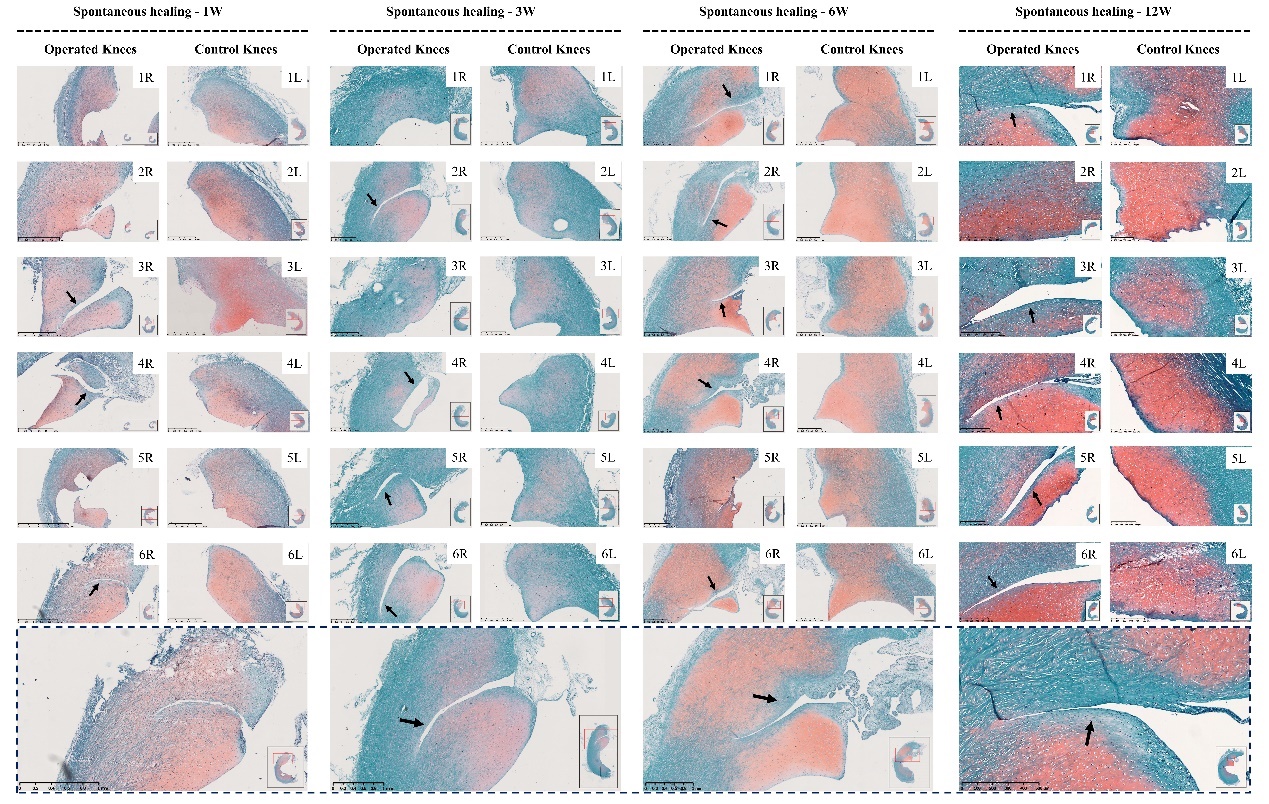


**Figure S3.** Safranin O-Fast green staining showing the GAG content of the experimental and contralateral native intact menisci in the immature group. The black arrows indicate fading of the Safranin O stain. The inferior pictures with a black dotted-line border indicate representative specimens in the corresponding observation period.


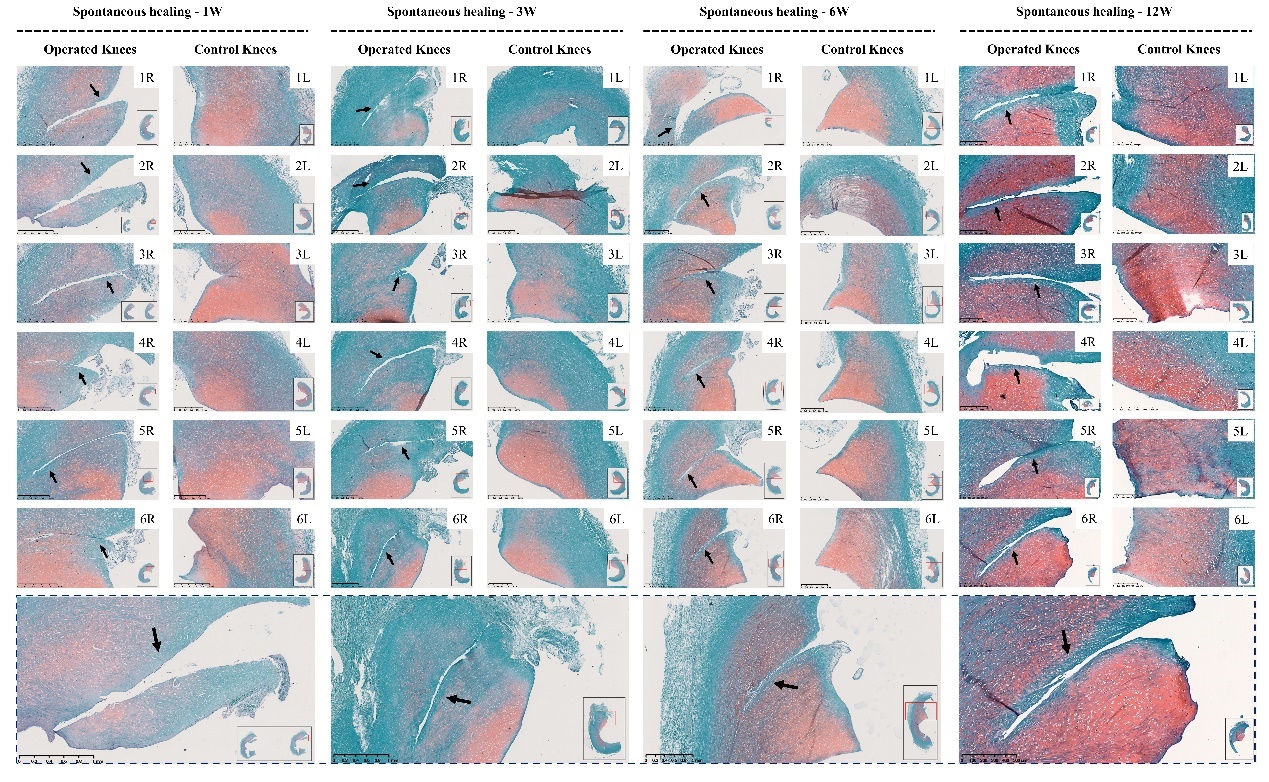


**Figure S4.** Safranin O-Fast green staining showing the GAG content of the experimental and contralateral native intact menisci in the mature group. The black arrows indicate fading of the Safranin O stain. The inferior pictures with a black dotted-line border indicate representative specimens in the corresponding observation period.


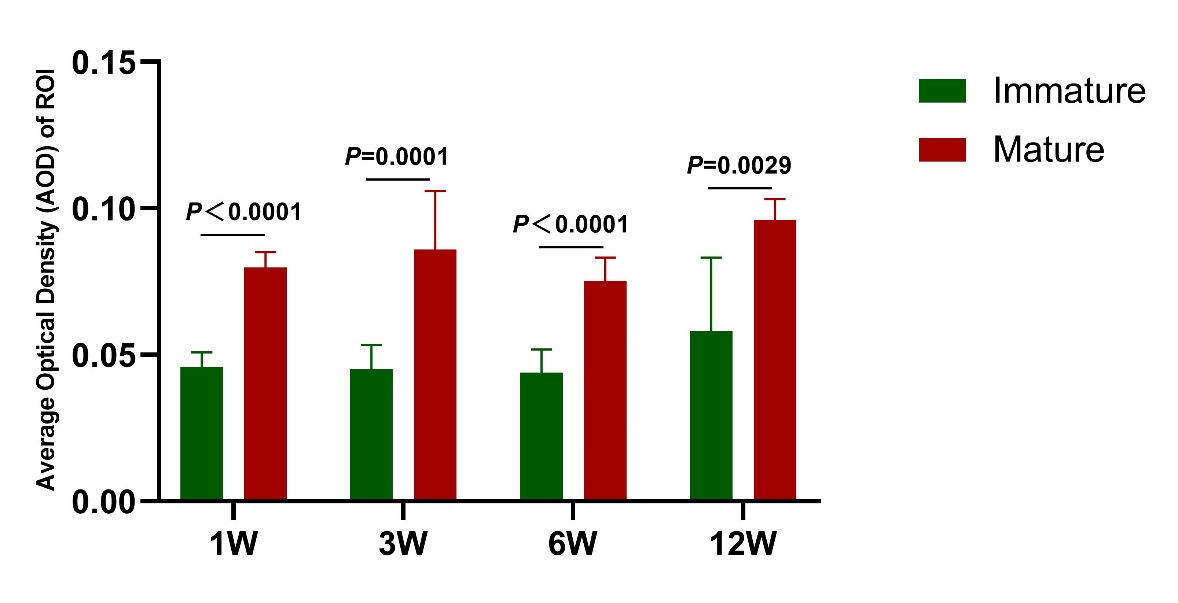


**Figure S5.** The comparative analysis of type 2 collagen immunohistochemistry between immature and mature menisci after untreated meniscal tears.


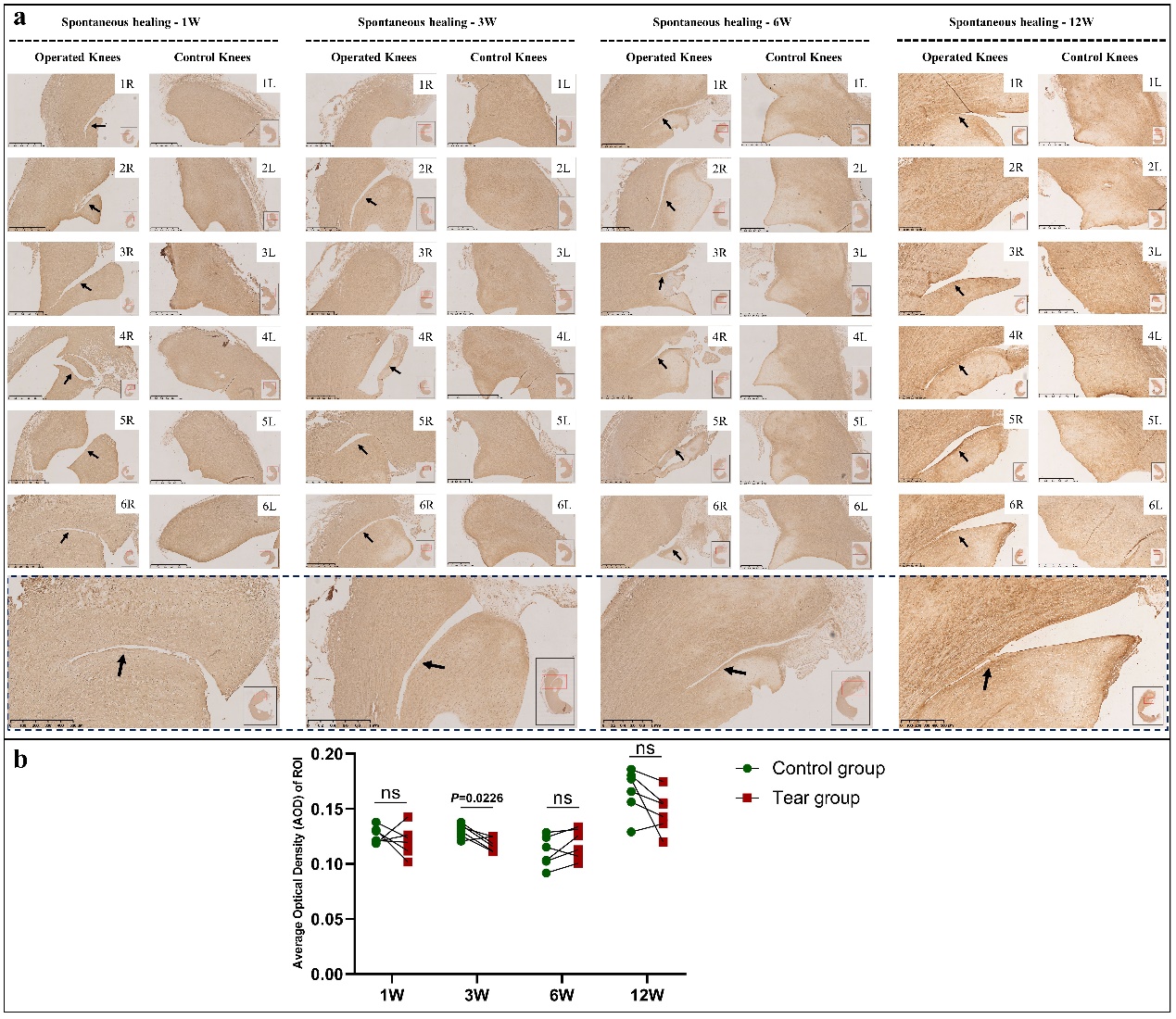


**Figure S6.** Immunohistochemical and semiquantitative evaluation of type 1 collagen content in immature menisci. (a) Immunohistochemical staining of type 1 collagen in the experimental and contralateral native intact menisci in the immature group. (b) Semiquantitative evaluation of type 1 collagen content within the ROI in immature menisci using AOD measurements. The black arrows indicate the tear site. The inferior pictures with a black dotted-line border indicate representative specimens in the corresponding observation period. AOD values are expressed as median values with 95% CIs. The sample size for each group was six**.** ns: no significant differences.


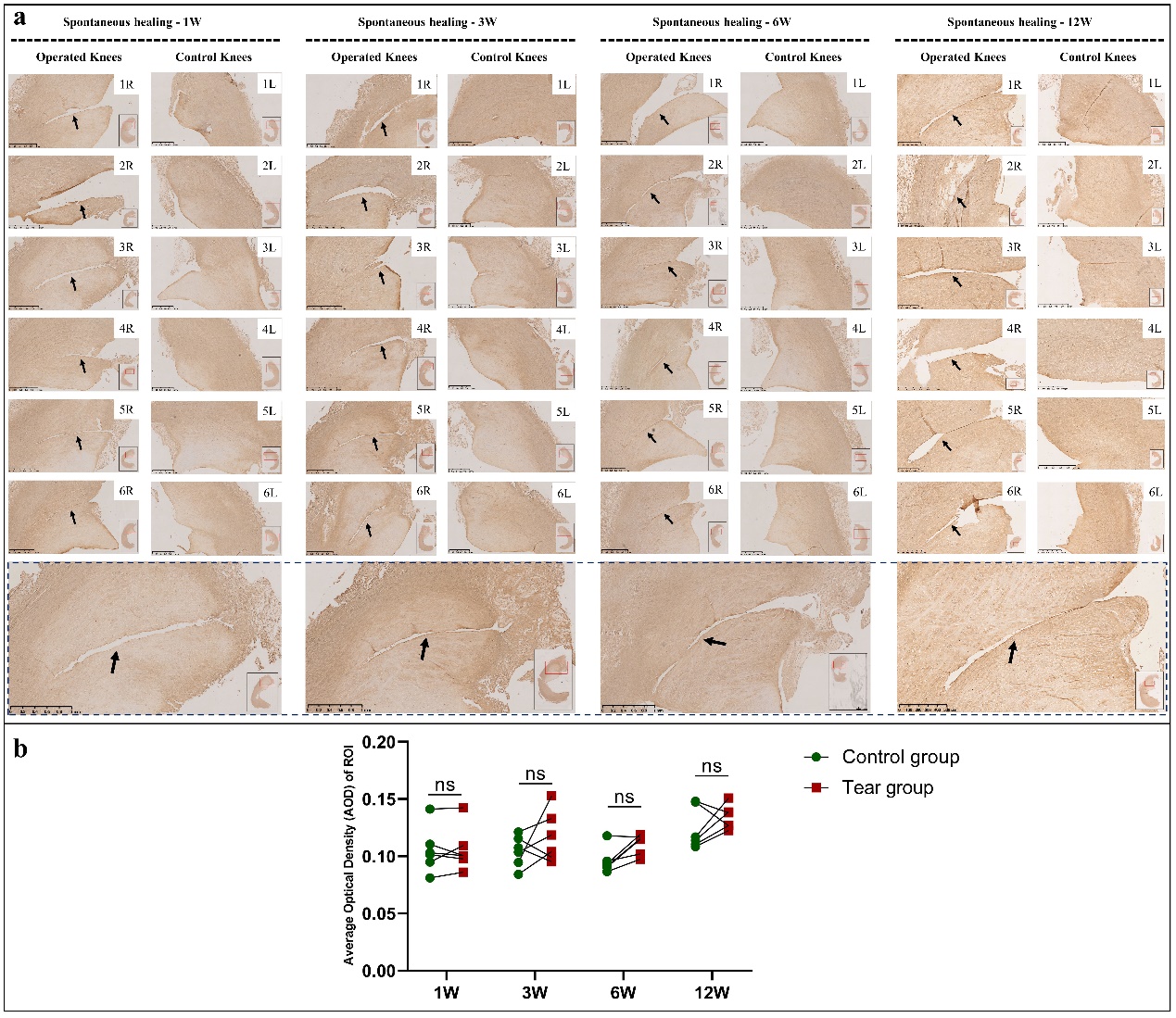


**Figure S7.** Immunohistochemical and semiquantitative evaluation of type 1 collagen content in mature menisci. (a) Immunohistochemical staining of type 1 collagen in the experimental and contralateral native intact menisci of the mature group. (b) Semiquantitative evaluation of type 1 collagen content within the ROI in mature menisci using AOD measurements. The black arrows indicate the tear site. The inferior pictures with a black dotted-line border indicate representative specimens in the corresponding observation period. AOD values are expressed as medians with 95% CIs. The sample size for each group was six.
